# American Veterinary Medicine1Response to a toast at the banquet given to Prof. A. Liautard, at the Twenty-fifth Anniversary of the American Veterinary College, Manhattan Hotel, New York, September 5, 1899.

**Published:** 1899-10

**Authors:** R. S. Huidekoper


### "AMERICAN VETERINARY MEDICINE."[1]

By Dr. R. S. Huidekoper.

Mr. Chairman and Gentlemen: The toast given to me to respond to—"American Veterinary Medicine"—is so closely associated with the man whom we meet to do honor to to-night that what I can say in response to it would be almost equally appropriate had the title been Alexander Liautard.

I ask you, gentlemen, to join me in a bumper to him, scientist, practitioner, teacher, friend, a native of the country which first gave birth to veterinary schools, and stands at the head of the veterinary profession to-day, and an adopted son of this our great country, which has been so much benefited by his sojourn among us, that his name will be an indelible landmark in American veterinary medicine.

Dr. Liautard, your health!

Any history of veterinary medicine in America is practically confined to the last half of this century, with the exception of an attempt made in 1806 by Dr. Benjamin Rush, the great physician, patriot, and statesman, who endeavored at that time to have a veterinary school added to the University of Pennsylvania. For the rest of the fifty years of the century we can find no trace of any advance being made. Early in the fifties Dr. George Dadd, a man who seems to have been of more than usual intelligence, appeared in Boston and organized an attempt at a veterinary school, and at the same time published, for a year or two, a veterinary journal. The school itself seems to have been soon abandoned, though the

[1] Response to a toast at the banquet given to Prof. A. Liautard, at the Twenty-fifth Anniversary of the American Veterinary College, Manhattan Hotel, New York, September 5, 1899.

books and seal of it passed to other hands and went to Philadelphia, and afterward into Ohio, and further West, where, on into the sixties, diplomas were issued bearing the forged signatures of men already dead, and others.

In 1857 an Act for the incorporation of the New York College of Veterinary Surgeons was signed at Albany, and an organization was commenced with Dr. John Busteed as professor of anatomy and surgery, but only a paper organization was kept up for several years, without any real work being done.

1862 is the year from which dates the commencement of a tangible effort in the establishment of a body of veterinarians who were to give to American veterinary medicine its first history. A number of veterinarians met in Philadelphia to perfect an Association which was the origin of what was to become the American Veterinary Medical Association of to-day. They purchased a minute-book, which I believe is still used by the Association, but a general quarrel seems to have taken place and the records of the first meeting were destroyed—you will find in the book to which I refer the stubs of the first pages which have been cut out.

On June 9, 1863, a meeting was called at the Astor House, in New York, which is generally now considered the first meeting of the Association. Among those present were William A. Wisdom, of Delaware; Dr. John Busteed, Alexander Liautard, R. S. Copeman, R. H. Curtis, A. Large, Wm. J. McCown, Louis Brandt, C. C. Price, W. Bannister, John Budd, E. Nostrand, Charles Burden, and James Mulligan, of New York; Charles M. Wood, E. M. Thayer, Wm. Saunders, R. Farley, J. H. Stickney, James Penniman, O. H. Flagg, and R. Wood, from Massachusetts; E. F. Ripley, from Maine; R. McClure, G. Mellor, J. C. Essenwein, E. H. Palmer, and Isaiah Michener, from Pennsylvania; G. W. Bowler, Ohio; Jacob Ditts, J. C. Higgins, W. R. Mankin, R. Jennings, A. Phillips, Jacob Phillips, J. C. Walton, A. C. Budd, and S. Humphrey, from New Jersey; and J. K. Quickfall and John Arnold, from London. This meeting seems to have given a more general interest and active start in veterinary affairs.

Drs. Rawson and Busteed and Alfred Roe, Esq., obtained a number of subscriptions, and, by arrangement with Dr. Liautard, leased his infirmary at 179 Lexington Avenue, for college and hospital purposes. On November 23, 1864 they opened the New York College of Veterinary Surgeons. Dr. Liautard was appointed superintendent of the hospital, and was the first professor of anatomy, operative surgery, and clinics.

Dr. James Robertson was one of the first graduates, and he became a member of the teaching faculty in 1868. In that year a reception was given to Prof. Gamgee, of London, who visited this country to investigate cattle diseases.

During this decade there were few graduate veterinarians in this country. Dr. Liautard held his diploma from the Imperial Veterinary Schools of France; Dr. Stickney, of Boston, had received his from the Royal Veterinary College, London; and the dozen others were foreigners, mostly Englishmen, with the Royal College degree. In 1875 the apple of discord again fell into the cage of the profession, and the faculty of the New York College resigned almost in a body and organized the American Veterinary College, which is now completing its quarter century of existence. This decade saw the establishment of the two veterinary schools in Canada and the founding of the *American Veterinary Review* by the United States Veterinary Association, with Prof. Liautard as its first editor.

Up to this time the meetings of the United States Veterinary Medical Association had been confined to Philadelphia, New York, and Boston. In the eighties more rapid strides were made. The United States Veterinary Medical Association extended its meetings to Cincinnati, Baltimore, and the West, securing a membership really more national in character. Three-year veterinary schools were established at Harvard and the University of Pennsylvania, and a number of veterinary schools sprang up through the West, in Ohio, Illinois, Iowa, Minnesota, Kansas, and elsewhere. The Bureau of Animal Industry was founded in 1884 under the strong guidance of Dr. D. E. Salmon, and, after exterminating contagious pleuro-pneumonia from the American continent, became a power which brought the value of the veterinarian before the public and gave new openings for a livelihood to the veterinary graduates of the new schools.

In 1880 the JOURNAL OF COMPARATIVE MEDICINE AND VETERINARY ARCHIVES was established. During the eighties a number of State veterinary and other local societies were organized, one of which, that of Pennsylvania, whipped and led by the untiring zeal of Dr. W. Horace Hoskins, has become a rival of the American Association in the value of the work it has done.

The last decade of the nineteenth century has seen a steady advance in the work of the previous ten years. The United States Veterinary Medical Association has spread its ægis still further West, and over the southern mountains of Tennessee, and it has extended its hand of professional brotherhood to Canada in the North, becoming the "American" instead of the "United States" Association. Most of the two-year schools of former days have increased their standard of education to the recognized minimum of a three-years' course.

Laws regulating the practice of veterinary medicine and establishing State boards of veterinary examiners have been enacted in a number of States, and show a good disposition on the part of the various communities, however stupidly and justly they may, like many other laws, err in their results.

This closing year of the century finds old differences healed here in New York City, and an amalgamation of the two colleges which separated twenty-five years ago, but which now put shoulder to shoulder under the new name of the New York American Veterinary College, or the Veterinary School of the New York University, with Professor Liautard still holding the reins. Now that unity has been established we will hope to see the Assembly at Albany give to the Metropolitan school the same financial support which it does to its rural sister in Ithaca.

In literature there has not been as much accomplished as might be hoped for. The *American Veterinary Review* and the JOURNAL OF COMPARATIVE MEDICINE AND VETERINARY ARCHIVES stand proud peers of their European colleagues, but their standard of excellence has been at the sore cost of labor and money of a few enthusiastic individuals. Some valuable scientific writings have emanated from the pens of employés of the Bureau of Animal Industry. Of practical veterinary works, Prof. Liautard stands at the head of the authors in numbers and value of his works.

Mr. Chairman and Gentlemen, I have occupied more time than I intended to do, and yet find I have only given a summary of what I would like to have said. I believe the data of my headings to be accurate and that a detailed history of American veterinary medicine during this century would be valuable and interesting if one would take the time to compile it. It has been a great pleasure to be at this meeting, and I thank you for your attention.

But, Mr. Chairman, I will detain you for one word more. I believe that I have forgotten myself as a factor in the making of the history of American veterinary medicine. When, as an assistant of Dr. Joseph Leidy, the anatomist, and Dr. D. Hayes Agnew, the surgeon, I took their advice, that the study of veterinary medicine opened up a field for a more interesting and broader scientific life than the routine of a simple human practitioner, and prepared myself to found the Veterinary Department of the University of Pennsylvania; when I broadened my conception of animal life by becoming a comparative anatomist instead of remaining a simple human anatomist; when I worked during two years in the laboratories of Nocard, Chauveau and Pasteur, Virchow and Koch and Ercolani, familiarizing myself with the bacteriological studies which led to our present knowledge of tuberculosis, glanders, and the other contagious diseases; when, as a teacher for twenty years, I found that *my* most interesting teacher and *my* most valued knowledge came from my study of comparative physiology, pathology, and hygiene; finally, when I added the diverse scientific knowledge given me by my study of veterinary medicine to the knowledge of human medicine which I had acquired at the University of Pennsylvania and in fifteen years of human practice, and had had the experience of fifteen years as a chief medical officer of the National Guard of Pennsylvania, in which I had charge of such bodies of men as the thousands remaining in the Conemaugh Valley after the Johnstown flood, and 7500 troops of Pennsylvania during the Homestead riots; then, gentlemen, I was amused, though some of my friends were annoyed, last year when I became the best advertised and the most universally damned "horse-doctor" in America.

The yelping jackal newspapers, playing upon the hysterical sentiment of the public, because they had no further sensational war news or other exciting information to furnish, and who wanted to commence an attack on the administration, had picked me, because I had widened my knowledge of medicine in learning the scientific truths of diverse animal life, instead of limiting it to that of one animal, man, and found opprobrium in the title of "horse-doctor." To me, personally, the attacks of that portion of the press was like water on a duck's back. I knew that I had the confidence and recognition of my work, which was all I desired, of the President, of the Secretary of War, of General Miles, and of my own commanding General, Major-General Brooke, now Governor-General of Cuba. I knew that the First Army Corps, of which I had the medical charge, had a better organization, better hospitals, more medical supplies, and, up to the time I left Chickamauga for Puerto Rico, a smaller sick-list and death-rate than any other corps of the army. All this is on record in the official papers in Washington and will be history when they are published. Therefore, I had no cause to regret that I was, and proud that I am, a veterinarian.

